# Alkaline phosphatase in pulmonary inflammation—a translational study in ventilated critically ill patients and rats

**DOI:** 10.1186/s40635-020-00335-x

**Published:** 2020-12-18

**Authors:** Jenny Juschten, Sarah A. Ingelse, Lieuwe D. J. Bos, Armand R. J. Girbes, Nicole P. Juffermans, Tom van der Poll, Marcus J. Schultz, Pieter Roel Tuinman, for the BASIC study investigators, F. M. de Beer, L. D. Bos, T. A. Claushuis, G. J. Glas, J. Horn, A. J. Hoogendijk, R. T. van Hooijdonk, M. A. Huson, M. D. de Jong, N. P. Juffermans, W. K. Lagrand, T. van der Poll, B. Scicluna, L. R. Schouten, M. J. Schultz, K. F. van der Sluijs, M. Straat, L. A. van Vught, L. Wieske, M. A. Wiewel, E. Witteveen

**Affiliations:** 1 Department of Intensive Care, Amsterdam University Medical Centers, location “VU”, Mail stop ZH 7D-172, De Boelelaan 1117, 1082 RW Amsterdam, the Netherlands; 2 Research VUmc Intensive Care (REVIVE), Amsterdam University Medical Centers, location “VU”, Amsterdam, the Netherlands; 3 Department of Intensive Care, Amsterdam University Medical Centers, location “AMC”, Amsterdam, the Netherlands; 4 Laboratory of Experimental Intensive Care and Anesthesiology (L⋅E⋅I⋅C⋅A), Amsterdam University Medical Centers, location “AMC”, Amsterdam, the Netherlands; 5 Emma Children’s Hospital–Pediatric Intensive Care Unit, Amsterdam University Medical Centers, location “AMC”, Amsterdam, the Netherlands; 6 Department of Pulmonology, Amsterdam University Medical Centers, location “AMC”, Amsterdam, the Netherlands; 7 Department of Intensive Care, OLVG hospital, Amsterdam, The Netherlands; 8 Division of Infectious Diseases, Amsterdam University Medical Centers, location “AMC”, Amsterdam, The Netherlands; 9 Center for Experimental and Molecular Medicine (CEMM), Amsterdam University Medical Centers, location “AMC”, Amsterdam, The Netherlands; 10 Mahidol–Oxford Tropical Medicine Research Unit (MORU), Mahidol University, Bangkok, Thailand; 11 Nuffield Department of Medicine, University of Oxford, Oxford, UK

**Keywords:** Alkaline phosphatase, Acute lung injury, AP, ARDS, Pulmonary inflammation, Mechanical ventilation

## Abstract

**Background:**

Alkaline phosphatase (AP), a dephosphorylating enzyme, is involved in various physiological processes and has been shown to have anti-inflammatory effects.

**Aim:**

To determine the correlation between pulmonary AP activity and markers of inflammation in invasively ventilated critically ill patients with or without acute respiratory distress syndrome (ARDS), and to investigate the effect of administration of recombinant AP on pulmonary inflammation in a well-established lung injury model in rats

**Methods:**

AP activity was determined and compared with levels of various inflammatory mediators in bronchoalveolar lavage fluid (BALF) samples obtained from critically ill patients within 2 days of start of invasive ventilation. The endpoints of this part of the study were the correlations between AP activity and markers of inflammation, i.e., interleukin (IL)-6 levels in BALF. In RccHan Wistar rats, lung injury was induced by intravenous administration of 10 mg/kg lipopolysaccharide, followed by ventilation with a high tidal volume for 4 h. Rats received either an intravenous bolus of 1500 IU/kg recombinant AP or normal saline 2 h after intravenous LPS administration, right before start of ventilation. Endpoints of this part of the study were pulmonary levels of markers of inflammation, including IL-6, and markers of endothelial and epithelial dysfunction.

**Results:**

BALF was collected from 83 patients; 10 patients had mild ARDS, and 15 had moderate to severe ARDS. AP activity correlated well with levels of IL-6 (*r* = 0.70), as well as with levels of other inflammatory mediators. Pulmonary AP activity between patients with and without ARDS was comparable (0.33 [0.14–1.20] vs 0.55 [0.21–1.42] U/L; *p* = 0.37). Animals with acute lung injury had markedly elevated pulmonary AP activity compared to healthy controls (2.58 [2.18–3.59] vs 1.01 [0.80–1.46] U/L; *p* < 0.01). Intravenous administration of recombinant AP did neither affect pulmonary inflammation nor endothelial and epithelial dysfunction.

**Conclusions:**

In ventilated critically ill patients, pulmonary AP activity correlates well with markers of pulmonary inflammation, such as IL-6 and IL-8. In animals with lung injury, pulmonary AP activity is elevated. Administration of recombinant AP does not alter pulmonary inflammation and endothelial or epithelial dysfunction in the acute phase of a murine lung injury model.

## Background

The dephosphorylating enzyme alkaline phosphatase (AP) can affect inflammation [1, 2]. AP attenuates the inflammatory response by successfully disarming lipopolysaccharide (LPS) from Gram-negative bacteria [3], and dephosphorylates extracellular proinflammatory adenosine triphosphate (ATP) to adenosine diphosphate (ADP), and subsequently to the anti-inflammatory and tissue-protective enzyme adenosine [4, 5]. In murine models of sepsis [6, 7], acute kidney injury (AKI) [8], and colitis [9], treatment with AP has been shown to lower the systemic inflammatory response and to protect against death [6, 7, 10, 11]. In a recent randomized clinical trial, systemic infusion of recombinant AP (recAP) improved renal function and prevented mortality in patients with sepsis-induced AKI [12, 13].

AP has been detected in lung tissue, though its exact origin remains unclear. Alveolar type II cells have been suggested as a potential source of AP [14, 15], but also neutrophils may exert a role due to the presence of AP in their secretory vesicles [16, 17]. The exact role and effects of AP in pulmonary inflammation is uncertain. Increased pulmonary AP activity has been demonstrated in animal models of acute lung injury [16, 18]. One clinical study showed elevated pulmonary AP activity in patients with chronic pulmonary disorders characterized by neutrophilic inflammation [19]. Treatment with AP yielded so far conflicting results on the pulmonary inflammatory response. One preclinical study showed administration of AP to increase the risk of developing acute lung injury [18], whereas another study suggests a protective role of AP in pulmonary inflammation [17].

The here presented translational study had two aims. First, it tested the hypothesis that pulmonary AP activity correlates with levels of markers of inflammation in bronchoalveolar lavage fluid (BALF) obtained from invasively ventilated critically ill patients. Second, it tested the hypothesis that administration of recAP diminishes pulmonary inflammation and endothelial and epithelial dysfunction in rats subjected to lung injury-induced intravenous injection of LPS combined with injurious ventilation.

## Methods

**Fig. 1:**
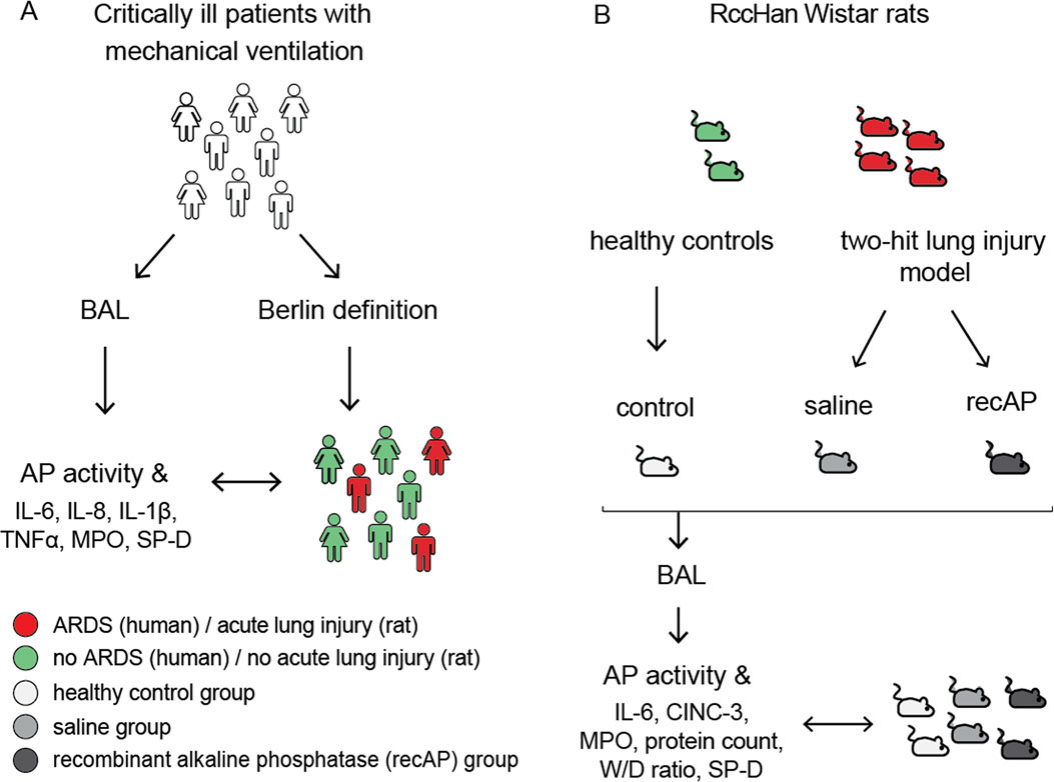
Schematic presentation of methodology for the clinical and preclinical study. **a** Clinical study. **b** Preclinical study. Abbreviations: ARDS, acute respiratory distress syndrome; AP, alkaline phosphatase; BAL, bronchoalveolar lavage; recAP, recombinant alkaline phosphatase; IL, interleukin; MPO, myeloperoxidase; SP-D, surfactant protein D; TNF, tumor necrosis factor; W/D ratio, lung wet-to-dry ratio

The clinical study was performed in invasively ventilated critically ill patients expected to stay in the intensive care unit (ICU) beyond the following day, with some of these patients having acute respiratory distress syndrome (ARDS) (Fig. 1a). In the preclinical study, RccHan Wistar rats were subjected to two pulmonary hits inducing lung injury, and some of the animals were treated with recAP (Fig. 1b).

### The clinical study

#### Study design and ethical considerations

The clinical investigation concerned a post hoc analysis of the “Biomarker Analysis in Septic Intensive Care Patients” (BASIC) study, a longitudinal cohort study conducted in the ICU of the Amsterdam University Medical Centers, location “AMC,” Amsterdam, the Netherlands. The investigational protocol was approved by the local institutional review board (METC 2010_335#B201112), and the study was registered at the Dutch Central Commission for Human bound Research (CCMO) (study identifier NL34294.018.10). Written informed consent was obtained before any study-related action took place.

#### Inclusion and exclusion criteria

Patients were included if (a) aged 18 years and older, (b) expected to stay at the ICU for more than 24 h, and (c) presenting with two or more SIRS criteria with or without suspicion of an infection. Patients were excluded if (a) treated with antibiotics > 48 h, (b) readmitted to the hospital, (c) included in another study targeting the inflammatory pathway, or (d) no written consent was obtained. For the current post hoc analysis, patients were also excluded if they had not received invasive ventilation.

#### Data collection, BALF, and blood collection

The database of the BASIC study contains baseline information including age, gender, reason for admission, type of admission (clinical, elective, or urgent surgery), illness severity (Acute Physiology and Chronic Health Evaluation [APACHE] IV score), 30-day mortality, and ICU and hospital length of stay. A dedicated team of trained researchers collected these baseline characteristics and outcomes and also re-scored presence of ARDS from previously applied AECC guidelines [20] to the current Berlin definition [21].

Within 48 h after start of invasive ventilation, a miniaturized bronchoalveolar lavage (BAL) was performed by inserting a standard 50 cm and 14-gage suctioning catheter via the endotracheal tube until resistance was encountered, and injecting 20 ml of saline over 4–5 s followed by immediate aspiration. At least 4 ml needed to be aspirated, and aliquots of BAL fluid (BALF) were processed and stored at − 80 °C. Before the BAL, blood was collected via the arterial line and centrifuged to obtain plasma, which was stored at − 80 °C.

#### Alkaline phosphatase activity

AP activity levels were determined in BALF as well as in plasma by a mammal-specific colorimetric alkaline phosphatase assay using p-nitrophenyl phosphate (pNPP) as a phosphate substrate (ab83369, Abcam; Cambridge, UK).

#### Markers of pulmonary inflammation and epithelial dysfunction

The inflammatory cytokines interleukin (IL)-6, IL-8, IL-1β and tumor necrosis factor (TNF)-α were measured in BALF using a cytometric bead array Flex Set multiplex assay according to the manufacturer’s instructions (B&D Biosciences, San Jose, California, USA). Myeloperoxidase (MPO), a specific marker of neutrophil activation, and surfactant protein D (SP-D), a marker of epithelial lung injury, were determined in BALF using human-specific enzyme-linked immunosorbent assay (ELISA) kits according to manufacturer’s instructions (Cayman Chemical, Ann Arbor, Minnesota, USA [for MPO]; HycultBiotech, Uden, The Netherlands [for SP-D]). Urea levels were assessed in BALF and plasma using quantitative colorimetric assay (BioAssay Systems, Hayward, CA).

#### Correction for dilution in human BALF samples

To correct measurements in BALF for the dilution factor induced by BAL, the ratio between urea in BALF and plasma was utilized as described before [22]. BALF was considered of insufficient quality if BALF urea levels were under the detection limit of 0.08 mg/dL or if the dilution factor was very high suggesting unreliable sampling. Those BALF samples were excluded from analysis*.* AP activity, as well as IL-6, IL-8, IL-1β, TNF-α, MPO, and SP-D levels were corrected for the dilution factor.

#### Endpoints

Endpoints of this part of the study were the correlations between pulmonary AP activity and levels of markers of lung inflammation and injury, such as IL-6, IL-8, IL-1β, TNF-α, MPO, and SP-D in BALF.

#### Power analysis

A formal power calculation was not performed. Instead, this post hoc analysis used BALF samples from all invasively ventilated patients included in the BASIC study. With the final sample size of 83 patients, a two-sided significance level of 0.05 and a power of 80% were obtained for a critical correlation coefficient (*r*) of 0.216.

#### Analysis plan

Continuous variables are presented as median with 25th–75th interquartile range (IQR) according to data distribution and categorical variables as absolute occurrences with percentages. Comparisons were performed using Mann–Whitney *U* or *χ*^2^ test, where appropriate.

The correlation between AP activity and IL-6 levels in BALF was assessed by Spearman’s correlation coefficient (rho, *r*), according to data distribution. This was repeated for the other inflammatory mediators. AP activity and inflammatory mediators were compared between patients with ARDS and those without ARDS. A correlation was considered strong if *r* ≥ 0.70, and moderate, weak, or negligible if *r* = 0.69–0.50, 0.49–0.30, and < 0.30, respectively [23].

A *p* value of < 0.05 was considered statistically significant. Statistical analyses were performed using the packages “*tidyverse*,” “*dplyr*,” and *“ggpubr*,” and figures were created with the package *“ggplot2*,” with R Studio interface (R core team. R: A Language and Environment for Statistical Computing. 2013. http://www.r-project.org/) from R Studio Interface.

### The preclinical study

This preclinical study follows the “Animal Research: Reporting of *In Vivo* Experiments” (ARRIVE) guidelines.

#### Two-hit lung injury model

The study used a well-established and frequently used rat model of lung inflammation induced by LPS *plus* injurious ventilation with a high tidal volume.

The experiment was conducted under protocols approved by the Animal Care and Use Committee of the Amsterdam University Medical Centers, location “AMC” (LEICA 132–AB and –AD). Animals were used in compliance with Institutional Standards for Use of Laboratory Animals, were handled 1 week before experiments to diminish stress activation, and were housed in a specific pathogen-free facility on a 12/12 h light/dark cycle with two rats per cage. Standard laboratory chow and water were available ad libitum. Experiments were spread over several weeks, and each day two rats were handled. The experiments took place in the Laboratory for Anesthesiology and Experimental Intensive Care (L·E·I·C·A) at the “AMC,” between 7:30 AM and 5:00 PM, and animal welfare was evaluated every 30 min until termination of the experiment. An online randomization generator was used to allocate animals to the three study groups.

In total, 21 specific pathogen-free male RccHan Wistar rats (Envigo, Horst, the Netherlands) with a mean ± SD body weight of 322 ± 19 g were used. One animal did not survive the challenge with LPS and 4 h of invasive ventilation due to prolonged hypotension that was non-responsive to fluid resuscitation. This animal was replaced to achieve a similar number of animals per group. Finally, eight rats received 1500 IU/kg recAP “recAP group”, which was kindly provided by AM Pharma (Bunnik, The Netherlands)—eight rats received normal saline as a placebo “saline group”—four rats served as controls and were left untouched “control group”. The recAP and saline group underwent the two-hit lung injury protocol, as described in detail previously [24]. The two-hit lung injury model consists of an intravenous injection of 10 mg/kg LPS (*Escherichia coli* serotype 0127:B8, Sigma Aldrich, St. Louis, MO, USA) followed by high tidal volume mechanical ventilation starting 2 h after LPS injection. Rats were subjected to ventilation in a volume-controlled mode with tidal volumes between 12 and 15 ml/kg and a positive end-expiratory pressure (PEEP) of 3.4 mbar (Babylog® 8000, Dräger, Germany). RecAP or saline was administered intravenously directly before start of high tidal volume ventilation. Adequate depth of anesthesia and analgesia was checked every 30 min with a short pain stimulus on a toe. Rats were ventilated for a total of 4 h after which they were sacrificed by exsanguination from the carotid artery, after a bolus of pentobarbital.

#### BALF and blood collection

Following exsanguination, the thorax was opened at the sternum and lungs with bronchi and trachea were resected in toto. The right main bronchus was clipped before flushing the left lung three times with 2 ml sterile saline. BALF was then centrifuged and stored at − 80 °C for further analyses. Arterial blood was drawn during exsanguination using a heparin-coated syringe, subsequently centrifuged, and plasma was obtained and stored at − 80 °C.

#### Alkaline phosphatase activity

AP activity was measured as described above for the clinical study.

#### Markers of pulmonary inflammation and endothelial and epithelial dysfunction

IL-6 and cytokine-induced neutrophil chemoattractant (CINC)-3 were measured in BALF using rat-specific ELISA kits (R&D systems, Minneapolis, MN, USA and Nordic Biosite AB, Täby, Sweden) according to manufacturer’s instructions. Myeloperoxidase (MPO) activity was measured in lung homogenate using a rat-specific ELISA kit (Hycult Biotech, Uden, the Netherlands). The total protein level in BALF was measured using the bovine serum albumin (BSA) Lowry method. By weighing the lower lobe from the right lung directly after excision and dividing it by the weight after 72 h in a stove with a temperature of 37 °C, the wet-to-dry (W/D) ratio was determined. Surfactant-associated protein D (SP-D) levels were determined in BALF using a rat-specific ELISA kit (Bio–Connect, Huissen, the Netherlands).

#### Endpoints

Endpoints of this part of the study were pulmonary levels of IL-6, CINC-3, MPO and SP-D, the lung wet-to-dry ratio, and BALF protein levels.

#### Power calculation

A sample size calculation indicated that eight animals per group was needed to detect a significant difference in IL-6 levels between intervention and control group, with a power of 0.8 with an effect size of 1.6 and a double signification level of 0.05.

#### Analysis plan

Comparisons between control and saline group, as well as saline and intervention group, were performed using Mann–Whitney *U* test, according to data distribution.

For this part of the study, a *p* value of < 0.05 is also considered statistically significant. The same software as described above was used for the statistical analyses and GraphPad Prism (Version 7.03, GraphPad Software, La Jolla, California, USA) for creation of figures.

## Results

### The clinical study

#### Patients

**Fig. 2:**
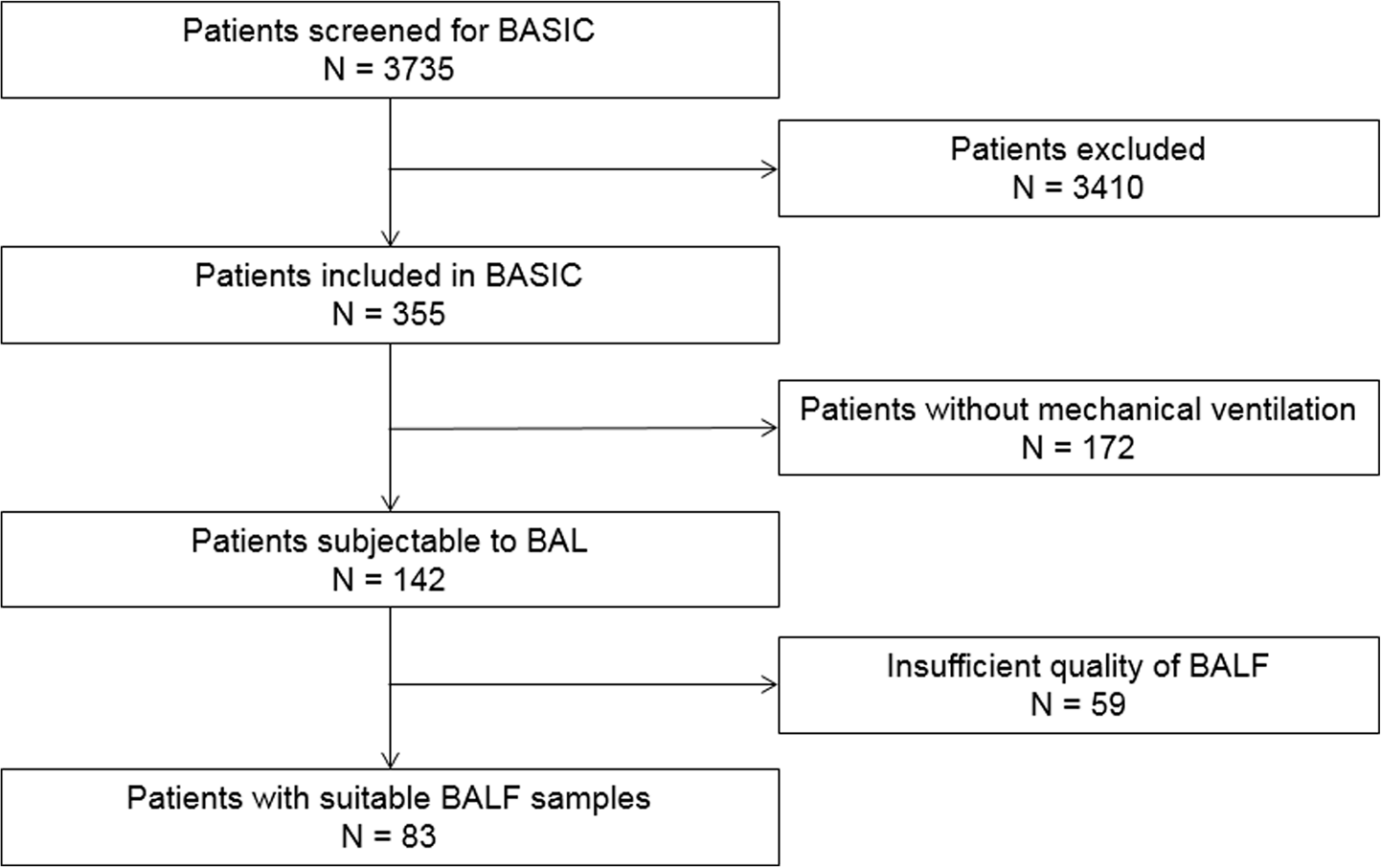
CONSORT diagram of the clinical study. Abbreviations: BASIC, “biomarker analysis in septic intensive care patients”; BAL, bronchoalveolar lavage; BALF, bronchoalveolar lavage fluid

The BASIC study included a total of 142 invasively ventilated critically ill patients. Patient flow is shown in Fig. 2. After excluding patients in whom BALF was considered of poor quality, 83 patients remained for the present analysis. Patient demographics are presented in Table 1. Patients were severely ill according to their APACHE IV scores, had a long length of ICU and hospital stay, and showed a high 30-day mortality rate. Only length of ICU stay was different between patients with ARDS and patients not having ARDS.

**Table 1:** Baseline characteristics of included patients on admission

Baseline characteristics	Patients with ARDS (*N* = 25)	Patients not having ARDS (*N* = 58)	*p* value
Age at admission (years)	61 (54–70)	63 (49–73)	0.739
Gender, male	12 (48)	38 (66)	0.161
Admission type			0.268
Medical	20 (80)	41 (71)	-
Surgical elective	2 (8)	2 (3)	-
Surgical emergency	3 (12)	15 (26)	-
APACHE IV score	81 (67–106)	72 (60–95)	0.266
PaO_2_/FiO_2_ ratio	161.5 (130.0–209.2)	248.5 (198.5–303.8)	< 0.001
30-day mortality	8 (32)	18 (33)	0.912
Hospital length of stay (days)	13 (6–13)	12 (6–23)	0.483
ICU length of stay (days)	4 (8–13)	3 (5–7)	0.017

For continuous variables data are presented as median and interquartile range () according to data distribution, and for categorical variables as numbers and percentage (%)

#### Correlation between pulmonary AP activity and inflammatory mediators

**Fig. 3:**
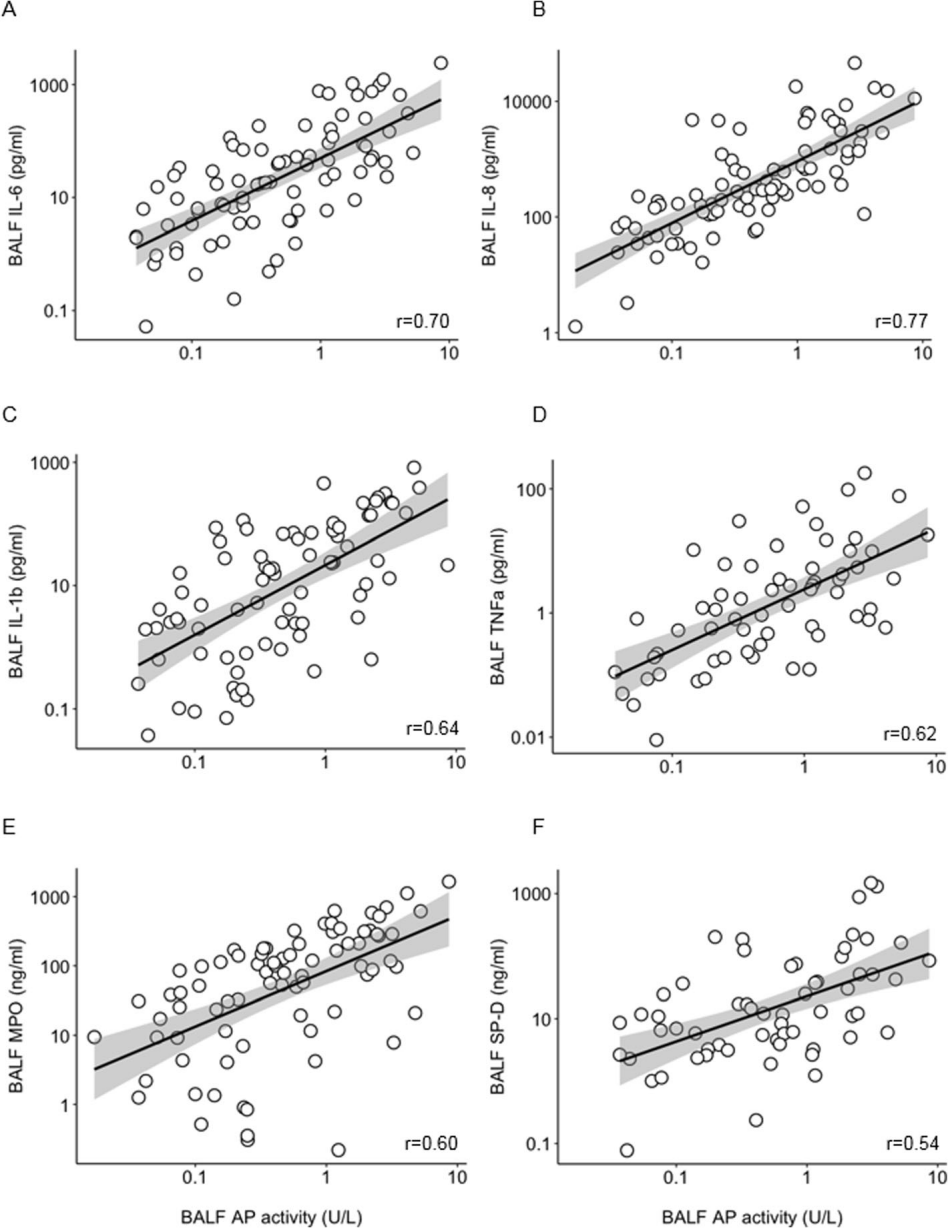
Correlations between AP activity and inflammatory mediators in BALF of invasively ventilated critically ill patients. **a** IL-6. **b** IL-8. **c** MPO. **d** IL-1β, **e** TNF-α. **f** SP-D. All levels are corrected for the BALF dilution factor. Abbreviations: AP, alkaline phosphatase; BALF, bronchoalveolar lavage fluid; IL, interleukin; MPO, myeloperoxidase; SP-D, surfactant protein D; TNF, tumor necrosis factor

**Fig. 4:**
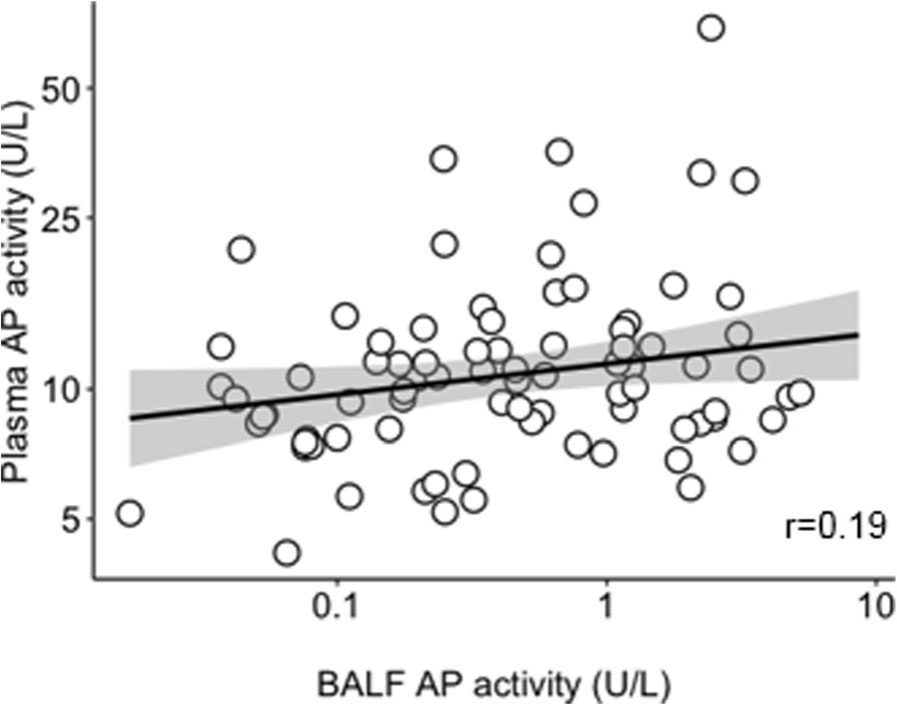
Correlation between pulmonary and systemic AP activity in invasively ventilated critically ill patients. Abbreviations: AP, alkaline phosphatase; BALF, bronchoalveolar lavage fluid

Pulmonary AP activity had a strong correlation with IL-6 levels in BALF (Fig. 3a). Likewise, pulmonary AP activity had a strong correlation with IL-8 levels in BALF (Fig. 3b). Pulmonary AP activity had a moderate correlation with IL-1β and TNF-α (Fig. 3 c and d), and with MPO and SP-D levels in BALF (Fig. 3 e and f). Pulmonary and systemic AP activity correlated poorly (Fig. 4). Pulmonary AP activity and levels of markers of inflammation were not different in patients with ARDS versus patients not having ARDS (Table 2).

**Table 2:** AP activity and markers of inflammation and lung injury in BALF, corrected for dilution factor

Measure	Patients with ARDS (*N* = 25)	Patients not having ARDS (*N* = 58)	*p* value
AP (U/L)	0.33 (0.14–1.20)	0.55 (0.21–1.42)	0.369
IL-6 (pg/ml)	46.57 (7.62–130.77)	17.55 (4.35–52.29)	0.128
IL-8 (pg/ml)	274.48 (108.96–3047.86)	341.91 (126.69–1369.8)	0.666
IL-1β (pg/ml)	17.91 (0.64–55.44)	10.54 (2.03–71.70)	0.603
TNF-α (pg/ml)	1.05 (0.19–7.09)	1.55 (0.44–5.00)	0.732
MPO (ng/ml)	30.62 (7.48–127.56)	98.60 (27.22–266.61)	0.061
SP-D (ng/ml)	14.45 (5.20–94.34)	11.35 (3.89–36.46)	0.308

Data is presented as median with IQR according to data distribution

*AP* alkaline phosphatase, *BALF* bronchoalveolar lavage fluid, *IL* interleukin, *MPO* myeloperoxidase, *SP-D* surfactant protein D, *TNF* tumor necrosis factor

### The preclinical study

#### Two-hit lung injury model

**Fig. 5:**
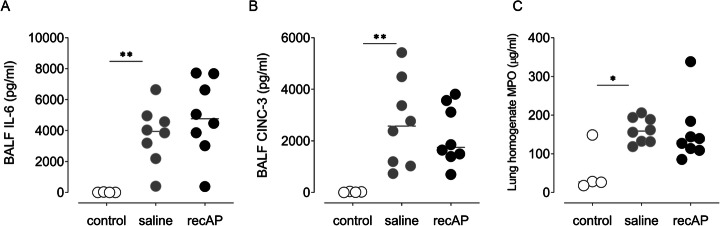
Markers of pulmonary inflammation in the two-hit lung injury model in rats. **a** IL-6 levels in BALF. **b** CINC-3 levels in BALF. **c** MPO levels in lung homogenate. Abbreviations: BALF, bronchoalveolar lavage fluid; IL, interleukin; CINC, cytokine-induced neutrophil chemoattractant; MPO, myeloperoxidase; recAP, recombinant alkaline phosphatase. *p value < 0.05; **p value < 0.01

**Fig. 6:**
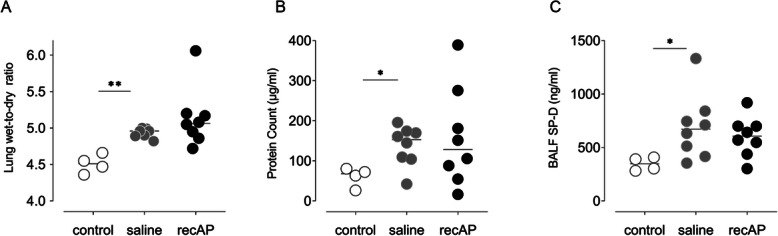
Markers of endothelial and epithelial barrier dysfunction in the two-hit lung injury model in rats. **a** Lung wet-to-dry ratio. **b** Total protein count in BALF. **c** SP-D levels in BALF. Abbreviations: BALF, bronchoalveolar lavage fluid; recAP, recombinant alkaline phosphatase; SP-D, surfactant protein D. *p value < 0.05; **p value < 0.01

The double hit resulted in pulmonary inflammation, as evidenced by markedly elevated levels of IL-6, CINC-3, and MPO in the lungs of rats in the saline group compared to control animals (Fig. 5). Endothelial and epithelial barrier dysfunction was also present, as evidenced by elevated lung W/D ratios, increased total protein levels, and increased SP-D levels in BALF (Fig. 6). Lung injury resulted in markedly increased pulmonary AP activity while systemic AP activity was not altered.

#### Effects of recAP

**Fig. 7:**
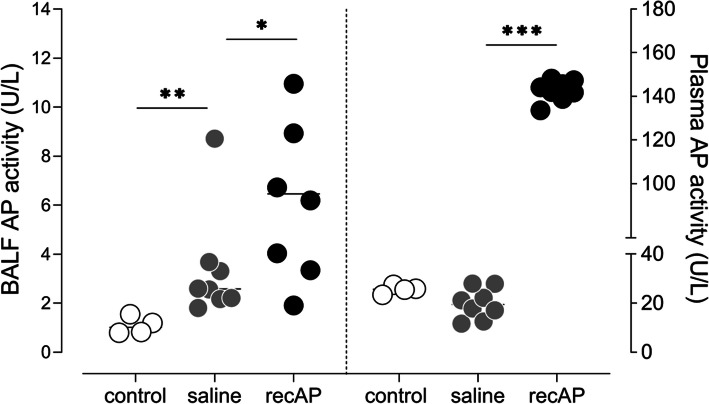
Pulmonary and systemic AP activity in the two-hit lung injury model in rats. Abbreviations: AP, alkaline phosphatase; BALF, bronchoalveolar lavage fluid; recAP, recombinant alkaline phosphatase. *p value < 0.05; **p value < 0.01; ***p value < 0.001

Intravenous administration of recAP resulted in higher AP activity, both within the pulmonary and systemic compartment (Fig. 7). In contrast to what was hypothesized, recAP administration did neither affect the pulmonary inflammatory response nor the alveolar–capillary permeability and epithelial dysfunction (Figs. 5 and 6).

## Discussion

In this cohort of invasively ventilated critically ill patients, AP activity correlated well with local levels of IL-6 and other proinflammatory mediators. Pulmonary AP activity was not different between patients classified as having ARDS and patients not having ARDS. The animal model confirmed the elevation of pulmonary AP activity along with increased levels of proinflammatory mediators and endothelial and epithelial dysfunction in the presence of lung injury. Recombinant AP administration did neither affect pulmonary inflammation nor endothelial and epithelial dysfunction in this model.

Our study is the first that measures pulmonary AP activity in invasively ventilated critically ill patients and determines the correlation with pulmonary inflammatory markers within the pulmonary compartment. It is also the first that translates this into a lung injury model in rats. AP administration was investigated in a clinically relevant and validated model mimicking sepsis-induced ARDS. The clinical part exhibits several strengths. The prospective character and a strictly followed analysis plan reduced bias, and the clear inclusion and exclusion criteria, of the BASIC study created a recognizable population of critically ill patients with high severity of illness scores. The number of included patients was large, and patients underwent a BAL relatively soon after start of invasive ventilation. The preclinical part was characterized by a well-established, and in our laboratory frequently used animal model reflecting clinically relevant lung injury. Systemic administration of recAP resulted in an elevation of pulmonary AP activity levels, showing that the drug reached the pulmonary compartment.

This study found a strong positive linear correlation between pulmonary AP activity and the inflammatory mediators IL-6 and IL-8, both playing pivotal role in neutrophil chemotaxis and apoptosis. Hence, the results of this study are in line with the suggestion that AP may exert a role in neutrophil-mediated pulmonary inflammation. In vitro, neutrophils increase AP expression in response to inflammatory stimuli, which is associated with enhanced chemotaxis, increased production of reactive oxygen species, and effective induction of apoptosis [17]. Also, several studies have shown increased AP expression by neutrophils in case of inflammation, for example in patients with bacterial infections [25–27] and chronic inflammatory conditions such as obesity [28].

The fact that pulmonary and systemic AP activity had a negligible correlation highly suggests that AP is produced within and stays within the pulmonary compartment. Alveolar type II cells have also been proposed to be a source of AP arguing that AP may function as a marker of epithelial injury [15, 29].. Indeed, pulmonary AP activity correlated with SP-D, a marker of epithelial injury, albeit this association was only moderate.

The lack of a difference in AP activity between patients with ARDS and patients not having ARDS may seem surprising. However, levels of pulmonary inflammatory mediators and clinical illness severity scores were also not different between both groups. The relatively small sample size or the fact that only critically ill patients with two or more SIRS criteria were included may attribute to this effect

AP administration did neither affect pulmonary inflammation nor endothelial or epithelial dysfunction in the preclinical model of lung injury. This contrasts, at least in part, the results of previous studies showing AP to have anti–inflammatory affects in models of sepsis [6, 7] and a model of AKI [8]. Two pathophysiological mechanisms are mainly held responsible for the detoxifying effects of AP in the sepsis models. First, the ability of AP to disarm LPS, and second, the ability to convert pro–inflammatory extracellular ATP into the anti-inflammatory and tissue-protective adenosine [13]. Elevated adenosine levels in BALF proved to reduce pulmonary inflammation in murine lung injury models showing beneficial effect of adenosine on the inflammatory response [30, 31]. However, in a murine influenza model, increased adenosine production resulted in progression from influenza to acute lung injury [32]. Administration of an AP inhibitor in this model decreased the inflammatory response, as well as leucocyte infiltration and epithelial dysfunction, but not the adenosine levels in BALF [18]. This may be attributed to the short half-life of adenosine or point to a different mechanism of action of AP inhibition, for example a reduced expression of AP in activated neutrophils.

Several weaknesses of the current study need to be addressed. With regards to the clinical study, it is important to realize that an additional control group, for example a group of less severe critically ill patients, or patients not under ventilation, is missing. Regarding the preclinical study, the animal model simulates lung injury within the acute phase, hence within the first 6 h after induction of lung injury. We cannot exclude the possibility that AP may have anti-inflammatory effects at later time points. We can also not exclude anti-inflammatory effects with higher dosages of AP, a local route of administration [33], or when AP would be administered before the challenge with LPS.

## Conclusion

In invasively ventilated critically ill patients, pulmonary AP activity correlates well with markers of inflammation suggesting a role of AP in neutrophil-mediated pulmonary inflammation. In animals with lung injury, induced by LPS and injurious ventilation, pulmonary AP activity is elevated. Additional AP administration does not affect pulmonary inflammation and endothelial and epithelial dysfunction in this model.

## Data Availability

Please contact author for data requests.

## Abbreviations

AP: Alkaline phosphatase
AKI: Acute kidney injury
APACHE: Acute Physiology and Chronic Health Evaluation
ARDS: Acute respiratory distress syndrome
ATP: Adenosine triphosphate
BAL: Bronchoalveolar lavage
BALF: Bronchoalveolar lavage fluid
BASIC: Biomarker analysis in septic intensive care patients
CINC: Cytokine-induced neutrophil chemoattractant
ELISA: Enzyme-linked immunosorbent assay
FiO_2_: Fraction of inspired oxygen
ICU: Intensive care unit
IL: Interleukin
LPS: Lipopolysaccharide
MPO: Myeloperoxidase
PaO_2_: Partial pressure of oxygen in atrial blood
PEEP: Positive end-expiratory pressure
recAP: recombinant alkaline phosphatase
SOFA: Sequential organ failure assessment
SP-D: Surfactant protein D
TNF: Tumor necrosis factor
W/D ratio: wet-to-dry ratio

## References

[1] Millan JL (2006). Alkaline phosphatases: structure, substrate specificity and functional relatedness to other members of a large superfamily of enzymes. Purinergic Signal.

[2] Rader BA (2017). Alkaline phosphatase, an unconventional immune protein. Front Immunol.

[3] Poelstra K, Bakker WW, Klok PA, Kamps JA, Hardonk MJ, Meijer DK (1997). Dephosphorylation of endotoxin by alkaline phosphatase in vivo. Am J Pathol.

[4] Peters E, Geraci S, Heemskerk S, Wilmer MJ, Bilos A, Kraenzlin B (2015). Alkaline phosphatase protects against renal inflammation through dephosphorylation of lipopolysaccharide and adenosine triphosphate. Br J Pharmacol.

[5] Tunjungputri RN, Peters E, van der Ven A, de Groot PG, de Mast Q, Pickkers P (2016). Human recombinant alkaline phosphatase inhibits ex vivo platelet activation in humans. Thromb Haemost.

[6] Su F, Brands R, Wang Z, Verdant C, Bruhn A, Cai Y (2006). Beneficial effects of alkaline phosphatase in septic shock. Crit Care Med.

[7] van Veen SQ, van Vliet AK, Wulferink M, Brands R, Boermeester MA, van Gulik TM (2005). Bovine intestinal alkaline phosphatase attenuates the inflammatory response in secondary peritonitis in mice. Infect Immun.

[8] Peters E, Ergin B, Kandil A, Gurel-Gurevin E, van Elsas A, Masereeuw R (2016). Effects of a human recombinant alkaline phosphatase on renal hemodynamics, oxygenation and inflammation in two models of acute kidney injury. Toxicol Appl Pharmacol.

[9] Tuin A, Poelstra K, de Jager-Krikken A, Bok L, Raaben W, Velders MP (2009). Role of alkaline phosphatase in colitis in man and rats. Gut..

[10] Bender B, Baranyi M, Kerekes A, Bodrogi L, Brands R, Uhrin P (2015). Recombinant human tissue non-specific alkaline phosphatase successfully counteracts lipopolysaccharide induced sepsis in mice. Physiol Res.

[11] Beumer C, Wulferink M, Raaben W, Fiechter D, Brands R, Seinen W (2003). Calf intestinal alkaline phosphatase, a novel therapeutic drug for lipopolysaccharide (LPS)-mediated diseases, attenuates LPS toxicity in mice and piglets. J Pharmacol Exp Ther.

[12] Pickkers P, Heemskerk S, Schouten J, Laterre PF, Vincent JL, Beishuizen A (2012). Alkaline phosphatase for treatment of sepsis-induced acute kidney injury: a prospective randomized double-blind placebo-controlled trial. Crit Care.

[13] Pickkers P, Mehta RL, Murray PT, Joannidis M, Molitoris BA, Kellum JA (2018). Effect of human recombinant alkaline phosphatase on 7-day creatinine clearance in patients with sepsis-associated acute kidney injury: a randomized clinical trial. Jama..

[14] Harada T, Koyama I, Shimoi A, Alpers DH, Komoda T (2002). Identification of pulmonary surfactant that bears intestinal-type and tissue-nonspecific-type alkaline phosphatase in endotoxin-induced rat bronchoalveolar fluid. Cell Tissue Res.

[15] Henderson RF, Scott GG, Waide JJ (1995). Source of alkaline phosphatase activity in epithelial lining fluid of normal and injured F344 rat lungs. Toxicol Appl Pharmacol.

[16] Bhalla DK, Gupta SK, Reinhart PG (1999). Alteration of epithelial integrity, alkaline phosphatase activity, and fibronectin expression in lungs of rats exposed to ozone. J Toxicol Environ Health A.

[17] Li H, Zhao Y, Li W, Yang J, Wu H (2016). Critical role of neutrophil alkaline phosphatase in the antimicrobial function of neutrophils. Life Sci.

[18] Woods PS, Doolittle LM, Hickman-Davis JM, Davis IC (2018). ATP catabolism by tissue nonspecific alkaline phosphatase contributes to development of ARDS in influenza-infected mice. Am J Physiol Lung Cell Mol Physiol.

[19] Cobben NA, Drent M, Jacobs JA, Schmitz MP, Mulder PG, Henderson RF (1999). Relationship between enzymatic markers of pulmonary cell damage and cellular profile: a study in bronchoalveolar lavage fluid. Exp Lung Res.

[20] Bernard GR, Artigas A, Brigham KL, Carlet J, Falke K, Hudson L (1994). The American-European consensus conference on ARDS. Definitions, mechanisms, relevant outcomes, and clinical trial coordination. Am J Respir Crit Care Med.

[21] Force ADT, Ranieri VM, Rubenfeld GD, Thompson BT, Ferguson ND, Caldwell E (2012). Acute respiratory distress syndrome: the Berlin definition. Jama..

[22] Rennard SI, Basset G, Lecossier D, O'Donnell KM, Pinkston P, Martin PG (1986). Estimation of volume of epithelial lining fluid recovered by lavage using urea as marker of dilution. J Appl Physiol.

[23] Mukaka MM (2012). Statistics corner: a guide to appropriate use of correlation coefficient in medical research. Malawi Med J.

[24] Juschten J, Ingelse SA, Maas MAW, Girbes ARJ, Juffermans NP, Schultz MJ (2019). Antithrombin plus alpha-1 protease inhibitor does not affect coagulation and inflammation in two murine models of acute lung injury. Intensive Care Med Exp.

[25] Karlsson A, Khalfan L, Dahlgren C, Stigbrand T, Follin P (1995). Neutrophil alkaline phosphatase activity increase in bacterial infections is not associated with a general increase in secretory vesicle membrane components. Infect Immun.

[26] McCall CE, Katayama I, Cotran RS, Finland M (1969). Lysosomal and ultrastructural changes in human “toxic” neutrophils during bacterial infection. J Exp Med.

[27] Sramkova L. Alkaline phosphatase in neutrophil leucocytes in infectious diseases. Acta Univ Carol Med (Praha). 1970:Suppl 43:1 + . 5502627

[28] Pan Y, Choi JH, Shi H, Zhang L, Su S, Wang X (2019). Discovery and validation of a novel neutrophil activation marker associated with obesity. Sci Rep.

[29] Hardaway RM (2006). A brief overview of acute respiratory distress syndrome. World J Surg.

[30] Hoegl S, Brodsky KS, Blackburn MR, Karmouty-Quintana H, Zwissler B, Eltzschig HK (2015). Alveolar epithelial A2B adenosine receptors in pulmonary protection during acute lung injury. J Immunol.

[31] Kohler D, Streienberger A, Morote-Garcia JC, Granja TF, Schneider M, Straub A (2016). Inhibition of adenosine kinase attenuates acute lung injury. Crit Care Med.

[32] Aeffner F, Woods PS, Davis IC (2014). Activation of A1-adenosine receptors promotes leukocyte recruitment to the lung and attenuates acute lung injury in mice infected with influenza a/WSN/33 (H1N1) virus. J Virol.

[33] Juschten J, Tuinman PR, Juffermans NP, Dixon B, Levi M, Schultz MJ (2017). Nebulized anticoagulants in lung injury in critically ill patients-an updated systematic review of preclinical and clinical studies. Ann Transl Med.

